# Associations of sedentary behaviours and incidence of unhealthy diet during the COVID-19 quarantine in Brazil

**DOI:** 10.1017/S1368980020004188

**Published:** 2020-10-22

**Authors:** André O Werneck, Danilo R Silva, Deborah C Malta, Crizian Saar Gomes, Paulo RB Souza-Júnior, Luiz O Azevedo, Marilisa BA Barros, Célia L Szwarcwald

**Affiliations:** 1 Department of Nutrition, School of Public Health, Universidade de São Paulo (USP), Av. Dr. Arnaldo, 715 - Cerqueira César, São Paulo 01246-904, Brazil; 2 Department of Physical Education, Federal University of Sergipe – UFS, São Cristóvão, Brazil; 3 Programa de Pós-Graduação em Saúde Pública, Escola de Enfermagem, Universidade Federal de Minas Gerais, Belo Horizonte, Minas Gerais, Brazil; 4 Instituto de Comunicação e Informação Científica e Tecnológica em Saúde (ICICT), Fundação Oswaldo Cruz (Fiocruz), Rio de Janeiro, Brazil; 5 Department of Public Health, School of Medical Sciences, Universidade Estadual de Campinas, Campinas, São Paulo, Brazil

**Keywords:** Sedentary behaviour, Feeding behaviour, Health behaviour, Social distancing

## Abstract

**Objective:**

Our aim was to analyse the association of change patterns on TV-viewing and computer/tablet use and incidence of elevated consumption of ultra-processed food consumption and lower consumption of fruits and vegetables during the COVID-19 pandemic.

**Design:**

Data of 39 208 Brazilian adults from a Behaviour Web Survey were used. Unhealthy nutrition habits were eating fruits or vegetables for <5 d/week and ultra-processed food (sugary foods, snacks, ready-to-eat frozen foods and embedded foods) for ≥5 d/week. For incidence indicators, we only considered participants without unhealthy behaviour before the quarantine. We created four categories of change in TV-viewing and computer/tablet use, considering a cut-off point of 4 h/d for each behaviour (1 – consistently low, 2 – become low during the quarantine, 3 – become high during the quarantine or 4 – consistently high). Analyses were adjusted for sex, age group, highest academic achievement, per capita income, working status during the quarantine, skin colour and adherence to the quarantine.

**Setting:**

Brazil.

**Participants:**

Brazilian adults (nationally representative).

**Results:**

Logistic regression models revealed that high TV-viewing and computer/tablet use incidence were associated with higher odds for elevated frequency of ultra-processed food consumption (TV-viewing: OR 1·70; 95 % CI 1·37, 2·12; computer/tablet: OR 1·73; 95 % CI 1·31, 2·27) and low consumption of fruit and vegetables (TV-viewing: OR 1·70; 95 % CI 1·29, 2·23; computer/tablet: OR 1·53; 95 % CI 1·08, 2·17) incidence. Consistent high computer/tablet use also presented higher odds for incidence of elevated frequency of ultra-processed food consumption.

**Conclusions:**

Participants with incidence of sedentary behaviours were also more likely to present incidence of unhealthy diet during the COVID-19 pandemic quarantine.

The pandemic of the new coronavirus disease (COVID-19) presented a fast spread worldwide and reached Brazil in last February (2020)^(1)^. The WHO has been recommending social distancing measures as quarantines aiming to reduce the velocity of transmission^(1)^. In this sense, the state health departments are strongly recommending social distancing, with a ‘stay at home’ message and restriction measures as closing schools and universities as well as non-essential commercial establishments since early March (2020).

Despite the benefits for the COVID-19 spread, the quarantine measures can be associated with the adoption of unhealthy behaviours, such as increasing sedentary behaviour and reduced diet quality, with increases in ultra-processed food consumption^(2,3)^. Sedentary behaviour, especially TV-viewing, is associated with increased energy intake^(4)^, which can be through different mechanisms as the higher consumption of ultra-processed foods while watching TV, as well as higher exposure to ultra-processed advertises^(5–7)^. In addition, other types of sedentary behaviours that become more frequent with social restriction measures (e.g. computer, tablet and smartphone) can also be associated with poor dietary habits given the new routine found during the quarantine.

However, the association between changes in sedentary/screen behaviours and incidence of poor dietary habits during the COVID-19 pandemic is unclear. In this study, we aimed at analysing the association of change patterns on TV-viewing and computer/tablet use and the incidence of elevated consumption of ultra-processed food consumption and lower consumption of fruits and vegetables during the COVID-19 pandemic. We hypothesise those participants with increased TV-viewing and computer/tablet use are more likely to also increase the frequency of ultra-processed food consumption and reduce the consumption of fruits and vegetables.

## Methods

### Sample

This was a national cross-sectional behavioural web survey, with retrospective information. Data collection was conducted between 24 April and 24 May 2020. The invitation of participants was through a chain sampling procedure. In the first stage, fifteen researchers involved in the study chose a total of 200 other researchers from different states in Brazil. Also, each researcher in the study chose twenty people from their social network, making a total of 500 people chosen. The people chosen in the first stage were called as seeds of the chain recruitment. These seeds sent the survey link to at least twelve people from their social networks, obeying a stratification by sex, age range (18–39; 40–59; 60+ years) and educational level (incomplete high school or less; complete high school or more). In addition, information about the Behaviour Survey was circulated through press releases, social communications from participating research institutions, state health departments and social media. All procedures were approved by the National Research Ethics Commission (CONEP) (process: 30598320.1.0000.5241). The total sample was composed of 45 160 participants. The sample was weighted (through the use of sampling weights) according to characteristics from the National Household Sample Survey (2019), considering the population in each state, education, age, sex and prevalence of chronic diseases, aiming to let the sample nationally representative.

### Ultra-processed food and fruit and vegetable consumption

Ultra-processed food and fruit and vegetable consumption were assessed by asking about the frequency of eating fruits, vegetables, sugary foods, snacks, ready-to-eat frozen foods and embedded foods. We classified risk behaviour as those reporting eating fruits or vegetables <5 d/week as well as eating at least one ultra-processed food (sugary foods, snacks, ready-to-eat frozen foods and embedded foods) 5 or more d/week, which were classified according to the NOVA classification^(8)^. For the purposes of this study, for ultra-processed food analysis, we only classified those without elevated frequency of ultra-processed food consumption before the quarantine, and for fruit and vegetable analysis, those without low frequency of fruit and vegetable consumption before the quarantine.

### TV-viewing and computer/tablet use

For TV-viewing, participants were asked: ‘Usually, before the pandemic, how many hours a day did you used to spend watching television?’ and ‘During the pandemic, how many hours a day have you been watching television?’. Possible answers for both are (1) none; (2) <1 h/d; (3) between 1 and <2 h/d; (4) between 2 and <3 h/d; (5) between 3 and <4 h/d; (6) between 4 and <5 h/d; (7) between 5 and <6 h/d; (8) 6 h/d or more. Also, computer/tablet use was assessed using two questions ‘Usually, before the pandemic, how many hours a day did you used to spend using a computer or tablet?’ and ‘During the pandemic, how many hours a day did you used to spend using a computer or tablet?’ with open answer. TV-viewing and computer/tablet use were classified using the cut-off point of 4 h/d in both moments (before and during the quarantine), especially considering the elevation in the incidence of CVD and mortality risk^(9–11)^. TV-viewing and computer/tablet use patterns were created using four change patterns: (1) Consistently low; (2) Become low (High to low); (3) Become high (Low to high) or (4) Consistently high.

## Covariates

We used sex, age group, highest academic achievement, working status during the quarantine, skin colour, diagnoses of COVID-19 on a close friend, co-worker or relative and adherence to the quarantine as covariates. The highest academic achievement was classified as incomplete high school, complete high school and college education or more. Per capita income before the pandemic was estimated through the total income of all members of the household divided by the number of members and classified as lower than one minimum wage (approximately 200 dollars), one to two minimum wages and three or more minimum wages. Working status during the quarantine was classified as currently not working, working on a normal routine and home office. Skin colour was classified as white or other. The adherence to the quarantine was classified as positive for those just going grocery stores and pharmacies or stayed strictly at home, leaving only for health care needs; and negative for adherence for those reporting that continued a normal life or tried to stay away from people, reducing contacts a little, not visiting the elderly, but kept working and leaving home as usual.

## Statistics

For the ultra-processed food and fruits and vegetable consumption analyses, proportion estimates and 95 % CI of all sample characteristics were calculated. Crude and adjusted models (adjusted for sex, age group, highest academic achievement, per capita income, working status, skin colour and adherence to the quarantine) were used to analyse the associations of TV-viewing and computer/tablet use patterns with incidence of elevated ultra-processed food consumption and low consumption of fruit and vegetables. All analyses were conducted using the software Stata 15.1.

## Results

The final sample was composed of 39 208 adults due to missing data. However, 5307 (weighted prevalence: 14·3 %) presented elevated frequency of ultra-processed food consumption and 26 572 (weighted frequency: 78·0 %) presented low consumption of fruit and vegetable before the pandemic and were excluded from the specific analyses. Therefore, the final sample was composed of 33 901 adults for ultra-processed food consumption and 12 636 adults for the low consumption of fruits and vegetables analysis. The samples for both analyses were similar, except for age group (sample of fruit and vegetable consumption analysis was slightly younger) and highest academic achievement (sample of fruit and vegetable consumption analysis presented slightly higher frequency of more than high school) (Table 1). The prevalence of participants who started consuming elevated ultra-processed food during the quarantine was 10·4 % (95 % CI 9·6, 11·3), while 17·6 % (95 % CI 15·9, 19·4) of participants started to have low consumption of fruit and vegetables during the quarantine (Table 1).

**Table 1:** Characteristics of the sample*

Variables	Ultra-processed food analysis (*n* 33 862)		Fruit and vegetable consumption analysis (*n* 12 618)	
	%	95 % CI	%	95 % CI
Sex (women)	52·7	51·0, 54·4	61·1	58·0, 64·1
Age group (years)				
18–39	47·1	45·5, 48·8	32·3	29·8, 35·0
40–59	34·8	33·3, 36·4	38·6	35·9, 41·4
60 +	18·1	16·7, 19·5	29·0	26·4, 31·8
Highest academic achievement				
No academic achievement or elementary school	9·7	8·6, 10·9	8·8	7·1, 10·9
High school	72·8	71·5, 74·0	66·7	64·4, 68·9
More than high school	17·5	16·9, 18·2	24·5	23·1, 26·0
Per capita income before the pandemic				
Lower than one minimum wage	49·4	47·7, 51·0	39·0	36·1, 42·0
1–2 minimum wages	24·8	23·3, 26·4	22·6	20·4, 25·0
2+ minimum wages	25·8	24·6, 27·1	38·3	35·7, 41·0
Working status during the quarantine				
No	52·7	51·1, 54·4	51·1	48·3, 54·0
Normal routine	21·3	19·9, 22·8	18·6	16·4, 21·0
Home office	25·9	24·6, 27·4	30·3	27·7, 33·0
Skin colour (non-white)	55·3	53·7, 56·9	44·1	41·2, 47·1
Adherence to the quarantine (yes)	73·3	72·7, 74·8	78·7	76·1, 81·1
TV-viewing patterns				
Consistently low	61·6	59·9, 63·2	65·6	62·8, 68·2
Become low	0·8	0·5, 1·1	1·1	0·5, 2·3
Become high	28·5	27·1, 30·0	25·7	23·4, 28·3
Consistently high	9·1	8·1, 10·3	7·6	6·2, 9·2
Computer/tablet use patterns				
Consistently low	33·7	32·2, 35·4	33·9	32·1, 36·6
Become low	4·0	3·4, 4·7	3·9	3·1, 4·8
Become high	21·1	19·8, 22·4	19·6	17·7, 21·7
Consistently high	41·2	39·6, 42·8	42·7	39·8, 45·6
Low consumption of fruit and vegetables during the quarantine	–		17·6	15·9, 19·4
Elevated ultra-processed food consumption during the quarantine	10·4	9·6, 11·3	–	

* Data are presented using values of frequency and 95 % CI.

Only participants who presented high TV-viewing incidence were at risk for elevated frequency of ultra-processed food consumption incidence (OR 1·70; 95 % CI 1·37, 2·12) and low consumption of fruit and vegetables incidence (OR 1·70; 95 % CI 1·29, 2·23) (Table 2). Participants with consistently high computer/tablet use (OR 1·58; 95 % CI 1·24, 2·01) and those becoming high computer/tablet use (OR 1·73; 95 % CI 1·31, 2·27) were at risk for elevated frequency of ultra-processed food consumption incidence, while only those with high computer/tablet use were at risk for low consumption of fruit and vegetables incidence when comparing with the consistently low computer/tablet use group (OR 1·53; 95 % CI 1·08, 2·17).

**Table 2:** Association of TV-viewing and computer/tablet use patterns with incidence of ultra-processed food and low fruit and vegetable consumption*

	Elevated frequency of ultra-processed food consumption		Low frequency of fruit and vegetable consumption	
	OR	95 % CI	OR	95 % CI
Crude model				
TV-viewing patterns				
Consistently low	REF		REF	
Become low	0·41	0·16, 1·04	0·70	0·18, 2·75
Become high	1·66	1·36, 2·04	1·73	1·33, 2·27
Consistently high	0·66	0·45, 0·95	0·72	0·43, 1·23
Computer/tablet use patterns				
Consistently low	REF		REF	
Become low	1·60	1·05, 2·44	1·49	0·90, 2·47
Become high	2·43	1·88, 3·13	1·86	1·34, 2·58
Consistently high	2·19	1·74, 2·75	1·14	0·84, 1·54
Adjusted model				
TV-viewing patterns				
Consistently low	REF		REF	
Become low	0·63	0·25, 1·61	0·97	0·27, 3·45
Become high	1·70	1·37, 2·12	1·70	1·29, 2·23
Consistently high	0·98	0·67, 1·44	0·90	0·52, 1·57
Computer/tablet use patterns				
Consistently low	REF		REF	
Become low	1·43	0·93, 2·20	1·34	0·79, 2·27
Become high	1·73	1·31, 2·27	1·53	1·08, 2·17
Consistently high	1·58	1·24, 2·01	1·96	0·69, 1·33

* Adjusted model: adjusted for sex, age group, highest academic achievement, per capita income before the pandemic, working status during the quarantine, skin colour and adherence to the quarantine.

## Discussion

Our main findings were that participants who started to present elevated TV-viewing were more likely to present incidence of elevated frequency of ultra-processed food consumption and low fruit and vegetable consumption. However, this association was not clear for considering the computer/tablet use patterns, in which participants who started to have computer/tablet high use and those with consistently high computer/tablet use presented higher odds for incidence of poor dietary habits.

The COVID-19 pandemic promoted several changes in health behaviours such as the reduction of physical activity, an increase in sedentary behaviour and an increase in the consumption of unhealthy foods^(3)^. However, our findings highlighted that people with high TV-viewing incidence were more likely to present incidence of unhealthy diet behaviours. The difference between computer/tablet use and TV-viewing can be in the co-occurrence of unhealthy foods such as snacks while watching TV^(5,6)^. Also, 14·2 % of the advertises of free-to-air TV channels from Brazil were food-related, and approximately 90 % of the food-related advertises were of ultra-processed food^(12)^, which can also lead to higher consumption of ultra-processed food as well as lower consumption of fruits and vegetables^(7)^. Given the negative effects of the sedentary behaviours and unhealthy diet for health, these findings highlight the need to identify and to intervene on population groups that presented higher incidence of more than one unhealthy behaviour during the COVID-19 pandemic, especially groups at risk for non-communicable diseases.

Our findings should be inferred in light of some limitations. Even with the weighting of our sample according to the National Household Sample Survey, the research had a low representativity of individuals with low socio-economic conditions, illiterate as well as people without access to the internet. Also, the retrospective design and self-reported questionnaires can be associated with recall bias. We were only able to analyse few types of ultra-processed food and only the frequency of consumption, without information on daily serving and specific energy contribution of each food, and we were not able to estimate the specific contribution of ultra-processed food on daily energy consumption. Moreover, the present research was conducted probably before the peak of COVID-19 cases and deaths in Brazil, which should be considered in the interpretation of our findings. On the other hand, we presented data of a large nationwide sample, which was weighted for being nationally representative assessing the associations between change in sedentary/screen behaviours and incidence of elevated frequency of ultra-processed food consumption and low consumption of fruit and vegetables during the COVID-19 pandemic quarantine and we consider this as a strength.

Thus, participants who started to present high TV-viewing and computer/tablet use during the COVID-19 pandemic quarantine were more likely to present incidence of elevated frequency of ultra-processed food consumption and low consumption of fruit and vegetables. Our study highlights that policies for the promotion of health behaviours during the COVID-19 pandemic should focus on multiple behaviours.
